# Microbial models with minimal mineral protection can explain long-term soil organic carbon persistence

**DOI:** 10.1038/s41598-019-43026-8

**Published:** 2019-04-25

**Authors:** Dominic Woolf, Johannes Lehmann

**Affiliations:** 1 Soil and Crop Science, Cornell University, Ithaca, NY 14853 USA; 2 Atkinson Center for a Sustainable Future, Cornell University, Ithaca, NY 14853 USA; 3 Institute for Advanced Study, Technical University Munich, Garching, Germany

**Keywords:** Carbon cycle, Microbial ecology, Carbon cycle

## Abstract

Soil organic carbon (SOC) models currently in widespread use omit known microbial processes, and assume the existence of a SOC pool whose intrinsic properties confer persistence for centuries to millennia, despite evidence from priming and aggregate turnover that cast doubt on the existence of SOC with profound intrinsic stability. Here we show that by including microbial interactions in a SOC model, persistence can be explained as a feedback between substrate availability, mineral protection and microbial population size, without invoking an unproven pool that is intrinsically stable for centuries. The microbial SOC model based on this concept reproduces long-term data (r^2^ = 0.92; n = 90), global SOC distribution (rmse = 4.7 +/− 0.6 kg C m^−2^), and total global SOC in the top 0.3 m (822 Pg C) accurately. SOC dynamics based on a microbial feedback without stable pools are thus consistent with global SOC distribution. This has important implications for carbon management, suggesting that relatively fast cycling, rather than recalcitrant, SOC must form the primary target of efforts to build SOC stocks.

## Introduction

Carbon (C) fluxes between the soil and atmosphere constitute a potentially large and uncertain source of carbon dioxide (CO_2_) emissions in response to rising global temperatures and land degradation. Conversely, soil organic carbon (SOC) is also receiving attention as a potential sink for atmospheric C through land-management practices that increase SOC stocks^1^. Predicting the impacts of environmental change or land management on SOC fluxes depends on the application of models. However, confidence in these predictions is hampered by the fact that the current generation of models do not represent the mechanistic processes that are known to occur, and also by the uncertainties in current models^2,3^. It has been argued that improving confidence in SOC projections requires a transition from first-order decay models to models that explicitly account for the activity of soil microbial communities^4–6^. Accordingly, a number of microbially-explicit SOC models have emerged in recent years^3,7–12^. Despite this recent interest in microbial models, traditional first-order decay models remain the mainstay of SOC modeling in most applications including Earth system models (ESMs), in part because microbial models have not yet demonstrated the reliability to provide robust predictions over long time scales and wide ranges of environmental conditions^12^.

SOC turnover has been modeled as a first-order decay process since at least 1945^13^. It was recognized early on that empirical fitting of a first-order model to SOC decomposition required multi-pool models in which different fractions of SOC decay with different mean residence times (MRTs)^14,15^. Such models are more than just an empirical convenience. They also reflect a conceptual paradigm that different “types” (in the broadest sense) of SOC have different representative MRTs. Interpreted in this way, a fraction of SOC appears extremely persistent, represented, for example, in the CENTURY model as a passive pool with an MRT of 400 to 2000 years^16^, or in the RothC model as an inert pool with an infinite MRT^17^.

Considerable effort has been expended in determining the physical or chemical characteristics that confer the presumed variation in MRT between pools. The dominant paradigm until the late 20^th^ century was that microbial decay products were more chemically recalcitrant than the parent organic matter^18^. More recently, it has become recognized that simple, readily-decomposable molecules are found within even the oldest SOC fractions, and that humic macromolecules were a product of extraction processes rather than existing *in situ*^4,19^. This observation, in combination with evidence that soil microorganisms can degrade any SOM, regardless of its chemical structure, when they can access it^20,21^, have eroded the chemical-recalcitrance paradigm in favor of a physical-protection paradigm^4,19^. The physical-protection concept proposes that SOC persistence is conferred by interactions with soil minerals, both by adsorption to reactive surfaces and by occlusion within aggregates^4,20,22–24^.

However, it remains unclear whether physical protection alone can fully explain the long persistence of SOC in slow-cycling pools. For example, there is a large discrepancy between the hundreds to thousands of years MRT of the most persistent pool in first-order models, and the turnover rate of soil aggregates, which have MRTs in the range of weeks to months^25,26^. Another cause for doubt stems from priming, whereby addition of a new C substrate increases (positive priming), or decreases (negative priming) the respiration rate of the already-existing SOC. Addition of fresh readily-metabolizable substrates typically cause positive priming^27,28^, except over short time frames where substrate switching can sometimes lead to negative priming as microbes switch their activity to metabolization of the new food source. While several mechanisms may contribute to priming^27^, the frequent observation of positive priming points to a single underlying fact—that the primed SOC was accessible to microbial decomposition, but its decomposition was previously limited by some factor that the new substrate was able to alleviate. Positive priming of subsoils by addition of fresh organic matter (FOM) is of particular relevance to the long-term stability of SOC^29^. Subsoil OC is usually considered to comprise mostly stabilized SOC, as indicated by its increasing radiocarbon age with depth^22,30^. Positive priming of subsoils thus involves the mineralization of old (and, therefore, generally assumed to be stabilized) SOC^28^. This is supported by observations that the ^14^C age of primed CO_2_ is comparable to that of the bulk SOC^29^, and that repeated FOM additions result in repeated priming rather than depleting a small more readily-primed SOC pool^31^. Thus, a substantial fraction of what is usually thought of as stabilized C is readily accessible to microbial mineralization once constraints on microbial population and activity are alleviated.

**Figure 1:**
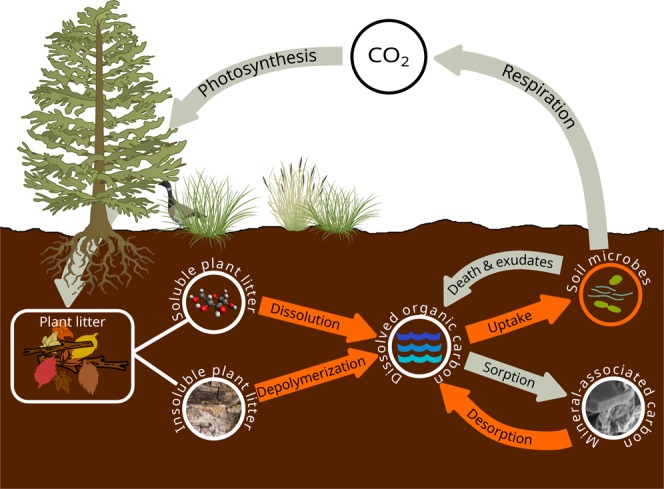
Schematic of microbial soil organic carbon (SOC) model SOMic 1.0. Carbon fluxes between pools are indicated by arrows, with fluxes whose rate constants are mediated by microbial enzyme activity indicated in orange.

Here we utilize a novel microbially-based SOC model (SOMic version 1.0) in which microbial interactions with mineral-associated organic matter remove the need for an intrinsically slow-cycling SOC pool, while still predicting long-term experimental data, and global SOC distribution. SOMic assumes that microorganisms take up only dissolved OC (DOC), because substrates must be in solution to cross the cell membrane (Fig. 1). Microbial uptake of DOC competes with sorption to minerals and occlusion within aggregates, whose rate is determined by mineral surface area (approximated by the clay fraction). Microbial uptake is then apportioned between growth and respired CO_2_ according to microbial C-use efficiency, which is dependent on temperature^8,32–34^. Organic matter inputs undergo depolymerization and/or dissolution before entering the DOC pool. Rates of depolymerization and dissolution of organic matter, and desorption of mineral-stabilized SOC are mediated by microbial enzyme activity according to reverse Michaelis-Menten dynamics^35^.

## Results

**Figure 2:**
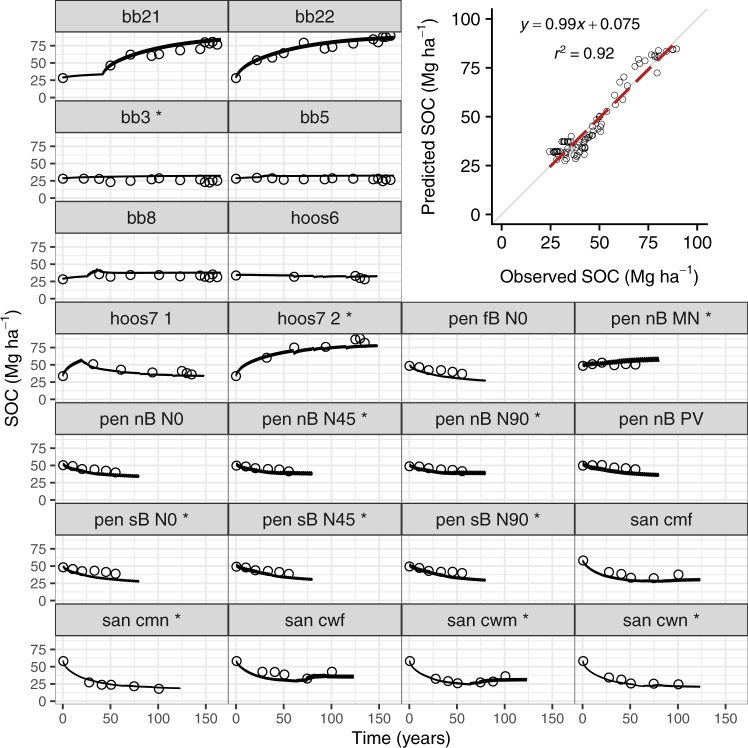
Soil organic carbon (SOC) stocks in the top soil horizon of twenty-two long-term agricultural experiments in Rothamsted, UK (“bb”, and “hoos” treatments), Pendleton OR, USA (“Pen”), and Sanborn MO, USA (“San”). Circles indicate observations, and lines the model predictions. SOC was predicted using the SOMic 1.0 model, as the sum of the five individual model pools (SPM, IPM, DOC, MB, and MAC). Calibration data are indicated with an asterisk (*) after the label. The inset panel of observed versus predicted values includes only data from the validation set (n = 90).

SOMic was calibrated and validated using 100–150 y time-series data from 22 long-term agricultural experiments (see Methods). The 22 sites were randomly assigned into 11 calibration sites and 11 validation sites, with the calibration and validation sets containing 90 and 75 data points, respectively (Fig. 2). Modeled SOC concentrations in the validation set correlated well with the observed values (r^2^ = 0.92, p < 0.001; Fig. 2). While this correlation is comparable to that achieved by traditional 1^st^ order models (for example RothC gives r^2^ = 0.92 when applied to the same validation set), it is notable that these results were obtained by SOMic without positing a highly stable SOC pool, thus demonstrating that the long-term dynamics of SOC can be explained by the interactions of microbial population size and activity with a mineral-associated organic matter pool that can cycle significantly faster than earlier models have required. The model pool with the longest base MRT (i.e., the MRT before it has been mediated by the microbial activity factor) is the mineral-associated carbon (MAC) pool, representing mineral-sorbed or -occluded SOC, whose base MRT varies from 5.5 y to 17 y for soil temperatures in the range 20–10 °C (significantly faster than the slow or passive pools in the CENTURY model^16^ which have MRTs of 20–50 y and 400–2000 y, respectively). Within the data set of these 22 long-term agricultural experiments, the microbial rate-modifying factor can increase the base MRTs by a factor of 1.05–2.8 (95% C.I., mean = 1.4), with the longest MRTs occurring at those locations and times with the lowest microbial population. The microbial carbon use efficiency (CUE) predicted by this calibration is 0.28 at 15 °C, with a temperature dependence of −0.0081 °C^−1^. These values compare well with literature values of CUE^33,34^ of 0.26–0.3 and its acclimated temperature dependency^32^ of −0.008 °C^−1^.

Our findings provide strong evidence to counter the assumption that mineral protection is the primary mechanism responsible for long-term (greater than decadal) SOC persistence. We have demonstrated that longer-term persistence may be rather be conferred by a combination of a competition between mineral-sorption and microbial uptake for available DOC, in combination with a microbial feedback whereby depleting the amount of easily metabolized organic matter leads to a decrease in microbial population and activity, which in turn lowers the rate at which remaining SOC is mineralized (Supplementary Information Figs 2 and 3). Conversely, the same mechanism in reverse allows the model to explain priming, as increasing the supply of fresh organic matter increases microbial population and activity, thus increasing the decomposition rate of other SOC pools. Although some previous studies have also proposed a similar role of microbial population and activity dynamics in SOC persistence and priming^10,29^, this is the first time that the mechanism has been demonstrated to agree well both with long-term (>100 y) observations and with global SOC distribution.

**Figure 3:**
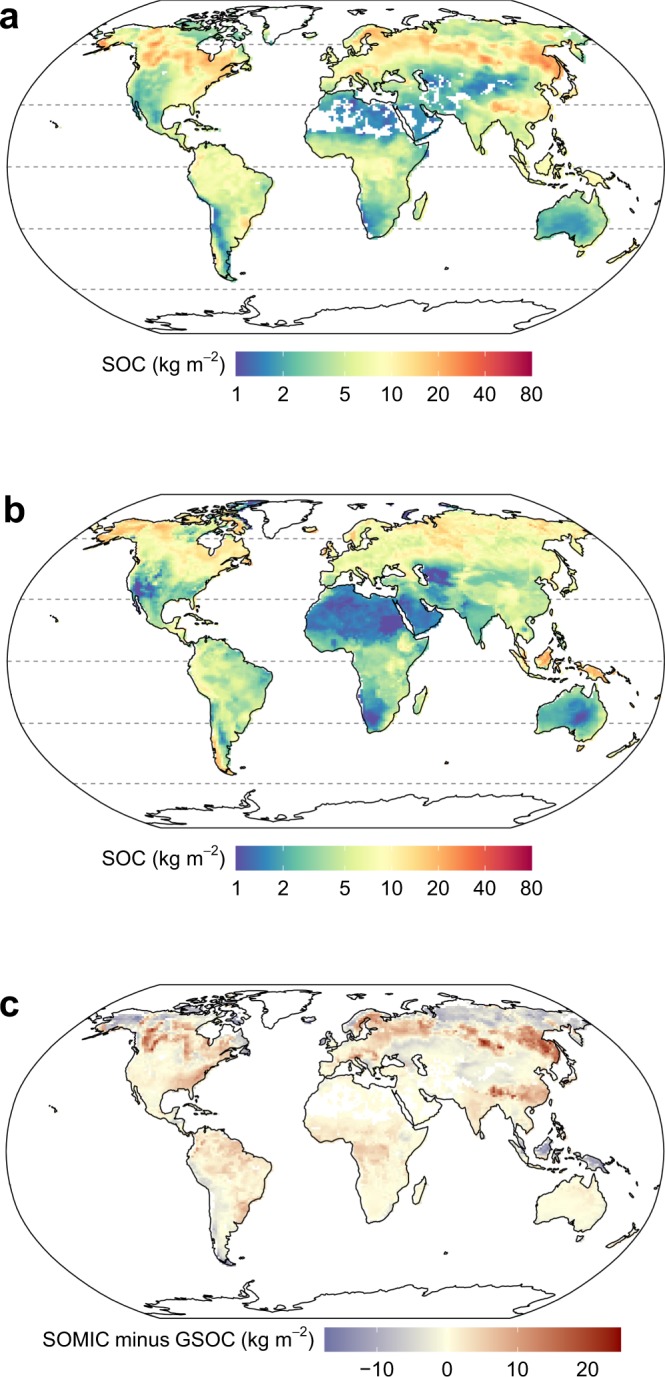
Global soil organic carbon concentration to 0–0.3 m depth (**a**) predicted by SOMic 1.0 (as the sum of the five individual model pools; SPM, IPM, DOC, MB, and MAC), forced with Community Earth System Model values for climate and litter inputs over the period 1800–2010, (**b**) from GSOC v1.0 provided as a reference comparison, and (**c**) the difference between them (i.e., SOMic minus GSOC; rmse = 4.7 kg C m^−2^).

**Figure 4:**
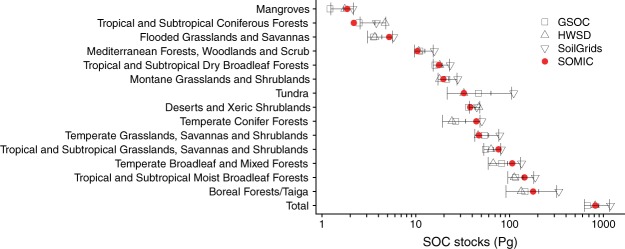
Global soil organic carbon (SOC), 0–0.3 m depth, disaggregated by biome. Red, filled circles indicate predicted values from SOMic 1.0 (as the sum of the five individual model pools; SPM, IPM, DOC, MB, and MAC). Open black symbols indicate estimates from GSOC (squares), the harmonized world soils database (HWSD; upward triangles), and SoilGrids (downward triangles). Error bars with ticks indicate the mean +/− 1 s.d. of GSOC, HWSD and Soil Grids.

To predict the global distribution of SOC in the top 0–0.3 m, the SOMic model was forced using the Community Earth System Model (Community Land Model (CLM) version 4.5) estimates of historic soil-temperature, soil-moisture, litterfall, and litter heterotrophic respiration from 1850 to 2010 (Supplementary Information Section 2.4) to predict the global SOC distribution (Fig. 3a). The global SOC distribution compares favorably with spatially-interpolated estimates of global SOC distribution such as the Global Soil Partnership’s Global Soil Organic Carbon (GSOC) map^36^ (Fig. 3b). The global SOC stocks predicted by SOMic using CLM forcing data are within the range of estimated values from an ensemble of GSOC, the harmonized world soils database^37^ (HWSD), and SoilGrids^38^ for all biomes, except tropical/subtropical coniferous forests where the SOMic estimate of 2.2 Pg C is slightly below the GSOC estimate of 2.5 Pg C (Fig. 4). Total global stocks of SOC in the top 0.3 m predicted by SOMic were 822 Pg C, which is also within the range of values derived from the HWSD (817 Pg C), GSOC (673 Pg C), SoilGrids (1190 Pg C), and the FAO/UNESCO Soil Map of the World^39^ (684–724 Pg C), (Fig. 4). These results demonstrate that SOMic provides robust estimates over a wide range of environmental conditions globally.

We have shown that models without slow or passive pools can explain most variation in SOC globally and over long times. However, a final question remains over whether such models are also compatible with the radiocarbon ages observed in soil profiles. Although some previous studies have found that microbial SOC models are compatible with the observed increasing ^14^C age of SOC with depth^10,40^, these have included mineral-stabilized pools with much longer mean residence times than those in SOMic (1150 y in ref.^10^, and 265 y in ref.^39^). Despite having only faster-cycling pools, when forced with vertical DOC advection rates from soil hydrology estimates of the CLM, SOMic also predicts radiocarbon age profiles closely aligned with observations (for example, predicted versus observed radiocarbon ages at Rothamsted, UK at depths to 0 to 0.9 m give an adjusted r^2^ = 0.67, p < 0.001, n = 78) (see Supplementary Information Section 3 and Supplementary Fig. 1).

## Discussion

Thus, we have shown that SOC models based on microbial population and activity dynamics, and without slow or passive pools, are compatible with observed SOC stocks, concentrations, distribution, and radiocarbon age. This does not *ipso facto* prove that such models are to be preferred over models that rely on slow and passive pools to reproduce the same observations. However, when considered in the context of observed priming of old SOC^29^, and relatively short aggregate turnover times^25^, this finding provides compelling support for the conjecture that we should rethink the balance of how we understand SOC persistence as being conferred to a lesser degree by mineral-stabilization and to a greater degree by ecological constraints than most previous models have assumed.

A recent inter-comparison study, which included four other microbial SOC models (CORPSE^41^, MIMICS^42^, MEND^43^, and RESOM^44^), has demonstrated that long-term model projections diverge depending on structural variations between models^45^. Important differences between models include the use of first-order, MM, reverse-MM, or equilibrium chemistry approximation kinetics; whether the model includes a DOC pool; whether microbial mortality rate is affected by MB density or soil moisture; texture-dependence of mineral protection; whether CUE varies with temperature or substrate; whether enzyme decomposition is included; and whether there exists a dormant microbial pool. The specific combination of structural features goes far to describing the differences between models, with alternative formulations representing alternative hypotheses that cannot yet be resolved^45^. Although the SOMic model described here differs from these other models in terms of its specific combination of features, it draws broadly from the same set of available hypotheses and assumptions summarized above. Distinctive features of the SOMic model are (1) that all biogeochemical processes depend on microbial activity (even sorption of DOC to minerals depends on the rate at which microbial activity creates DOC to sorb and the rate at which microbial uptake competes for DOC), and (2) that the desorption of mineral-protected SOC in SOMic has a base rate constant that is one to two orders of magnitude faster than in previous models which have predicted radiocarbon ages of subsoils in line with observations^10,40^. In addition to the structural variation between existing models, there are a number of potential model features which are not yet represented in any of these models, but which can be expected to further improve model performance over a broad range of environmental conditions and timescales. Additional features that may be expected improve performance of future generations of model include, for example, soil mineralogy^46^, mesofauna^47^, pH^48^, nutrient stoichiometry^49^, plant-microbe interactions^50^, and how the functional composition of the microbial community varies in response to factors like stress and substrate abundance^51^. Informing future model development by further inter-comparison studies (for example looking at diverging model performance over a range of timescales from hours to centuries), in combination with a process of incorporating further processes that are not yet well represented, can be expected to lead to a rapid improvement in SOC models over the coming years.

In conclusion, the finding that long-term SOC persistence arises from microbial interactions with mineral-associated carbon, rather than from intrinsic resistance of the SOC to decomposition, has important implications for the management of SOC. This is particularly relevant in the context of the rapidly growing interest in SOC’s potential role in climate-change mitigation, as for example exemplified in the 4 per mille initiative^52^. It has generally been assumed that recalcitrant carbon should form the primary target of efforts to build SOC stocks. Whereas, this new understanding suggests that management efforts to increase SOC will need to adapt by instead targeting the relatively fast cycling SOC that makes up the majority of SOC.

## Methods

### The SOMic v.1.0 model defines five SOC pools

- *C*_1_ is the carbon in readily soluble plant matter (SPM).
- *C*_2_ is the carbon in insoluble plant matter (IPM).
- *C*_3_ is dissolved organic carbon (DOC).
- *C*_4_ is mineral-associated organic carbon (MAC).
- *C*_5_ is the carbon in living microbial biomass (MB).

Inputs of fresh plant litter (L) to the soil are divided between SPM and IPM according to the readily-soluble fraction (*f*_*s*_). Decomposition products of all the pools enter the DOC pool (C_3_) before they are either taken up by microbes, or sorbed to minerals. Each of the pools (*C*_1_ to *C*_5_) has an associated decomposition rate factor (*k’*_1_ to *k’*_5_), respectively. The decomposition rate factors *k’*_1_ to *k’*_5_ are not constants, but rather are modified from each pool’s base rate constant (*k*_1_ to *k*_5_) by rate-modifying coefficients that vary dynamically over time. All rate factors have rate-modifying coefficients for temperature and moisture. All decomposition rate factors except *k’*_5_ (for microbial biomass turnover) are also modified by a microbial rate-modifying coefficient derived from reverse Michaelis-Menten (MM) kinetics^35^. Turnover of microbial biomass is assumed to be first-order with microbial biomass, and not modified by reverse-MM kinetics. The rate factor (*k’*_3_) for removal of C from the DOC pool is also dependent on competition between microbes and mineral sorption, where *f*_*sorb*_ is the fraction of C removed from the DOC pool that is sorbed to minerals. The sorption affinity of minerals for DOC is assumed to be a linear function of soil clay content. Microbial carbon uptake is partitioned between growth and respiration according to the microbial carbon use efficiency, which varies linearly with temperature.

The rates of change of carbon in each pool and the rate of CO_2_ efflux are shown in differential form below (Equations 1 to 6). Note that these equations are not first-order reactions, because the rate factors *k*′_1_ to *k*′_5_ are themselves functions of microbial biomass abundance through reverse-MM kinetics. A detailed description of how the various parameters and factors in the model were derived and calibrated is given in the Supplementary Information (Supplementary Methods).

1 $$\frac{d{C}_{1}}{dt}={f}_{s}\frac{dL}{dt}-{k}_{1}^{^{\prime} }{C}_{1}$$

2 $$\frac{d{C}_{2}}{dt}=(1-{f}_{s})\frac{dL}{dt}-{k}_{2}^{^{\prime} }{C}_{2}$$

3 $$\frac{d{C}_{3}}{dt}={k}_{1}^{^{\prime} }{C}_{1}+{k}_{2}^{^{\prime} }{C}_{2}+{k}_{4}^{^{\prime} }{C}_{4}+{k}_{5}^{^{\prime} }{C}_{5}-{k}_{3}^{^{\prime} }{C}_{3}$$

4 $$\frac{d{C}_{4}}{dt}={f}_{sorb}{k}_{3}^{^{\prime} }{C}_{3}-{k}_{4}^{^{\prime} }{C}_{4}$$

5 $$\frac{d{C}_{5}}{dt}={\rm{CUE}}(1-{f}_{sorb}){k}_{3}^{^{\prime} }{C}_{3}-{k}_{5}^{^{\prime} }{C}_{5}$$

6 $$\frac{d{{\rm{CO}}}_{2}}{dt}=(1-{\rm{CUE}})(1-{f}_{sorb}){k}_{3}^{^{\prime} }{C}_{3}$$

### Statistical methods

All summary statistics (r^2^, rmse, p-values, means and standard deviations) were calculated using base functions of the R programming language. The estimated uncertainty in rmse, expressed as 1 s.d. of the error, was calculated using Monte Carlo bootstrapping^53^, with 10^4^ iterations and a sample size of n = 50 with replacement.

## Supplementary information

Microbial models with minimal mineral protection can explain long-term soil organic carbon persistence: Supplementary Information

## Data Availability

All data used in model calibration and validation are summarized in the Supplementary Information, with links provided to the repositories from which the original data can be accessed. The code for the SOMic model can be accessed at https://github.com/domwoolf/somic1.
