# Supplementary material for: Microbial models with minimal mineral protection can explain long-term soil organic carbon persistence
Source: Sci Rep. 2019 Apr 25;9:6522. doi: 10.1038/s41598-019-43026-8 (PMC6484006; doi:10.1038/s41598-019-43026-8)
Supplement: Supplementary file 1 — Microbial models with minimal mineral protection can explain long-term soil organic carbon persistence: Supplementary Information [file 41598_2019_43026_MOESM1_ESM.pdf]

Microbial models with minimal mineral protection can explain long-term soil organic carbon persistence

Supplementary Information

Dominic Woolf[1,2,*] and Johannes Lehmann[1,2,3]

[1]Department of Soil and Crop Science, Cornell University, Ithaca, NY 14853, USA.
[2]Atkinson Center for a Sustainable Future, Cornell University, Ithaca, NY 14853, USA.
[3]Institute for Advanced Study, Technical University Munich, Garching, Germany.
[*]d.woolf@cornell.edu

1 Supplementary Methods

1.1 Model pools

The SOMic model defines five organic carbon pools:
C_1: Soluble plant matter (SPM)
C_2: Insoluble plant matter(IPM)
C_3: Dissolved organic carbon (DOC)
C_4: Live microbial biomass (MB)
C_5: Mineral-associated organic carbon (MAC)

1.2 Partitioning of organic matter inputs

Organic matter inputs are divided into readily-soluble (SPM) and insoluble (IPM) fractions by a ratio (SPM/IPM) that depends on the biomass type. In SOMic version 1.0 the ratio is based on the default ratio of decomposable to resistant plant material in the RothC 26.3 model[1]. This gives default values for SPM/IPM of 1.44 for agricultural crops and improved grassland, 0.67 for unimproved grassland and scrub (including Savanna), and 0.25 for woodland and forest.

1.3 Decomposition of organic matter inputs

Carbon from from both the readily-soluble (SPM) and insoluble (IPM) plant matter pools is transferred to the dissolved organic carbon (DOC) pool, although at different rates. Before entering the DOC pool, IPM must be depolymerized by microbial exoenzymes. Therefore, the rate at which IPM carbon transfers to DOC is mediated by the size of the microbial biomass (MB) pool. Reverse Michaelis-Menten (MM) kinetics (Section 1.4.4) were used to model the relationship between microbial biomass and depolymerization[2]. It was assumed that microbial enzyme activity also mediates the rate at which SPM is transferred to DOC, based on observed positive correlations between microbial activity and short-term DOC leaching from litter[3–5], driven by mechanisms including the microbial depolymerization of non-structural carbohydrates and phenolics[6], and release of SPM from the structural matrix as structural components are themselves degraded[3,5,7]. Accordingly, transfer of SPM to DOC was also modelled using reverse MM kinetics. Decomposition rates are also assumed to vary with soil temperature, moisture, and vegetation cover. The quantity of SPM_i and IPM_i in a time period i is then given by the quantities of SPM_{i-1} and IPM_{i-1}, respectively, in the previous time period, plus the amount of organic carbon inputs added during the current time period, according to

$$\mathrm{SPM}_i = \mathrm{e}^{-\tau\theta\nu\mu k_s}\,\mathrm{SPM}_{i-1} + f_s \mathrm{AOC}_i, \tag{1}$$

and

$$\mathrm{IPM}_i = \mathrm{e}^{-\tau\theta\nu\mu k_i}\,\mathrm{IPM}_{i-1} + \left(1 - f_s\right) \mathrm{AOC}_i, \tag{2}$$

where, τ is a rate modifying factor due to temperature, θ is a rate modifying factor due to soil moisture, ν is a rate modifying factor due to plant cover, μ is a rate modifying factor due to microbial biomass; k_i and k_s are the base rate constants for IPM and SPM decomposition, respectively, before the rate-modifying factors have been applied; AOC_i is the organic carbon added to the soil in time period i, and f_s is the soluble fraction of the added organic carbon.

1.4 Calculation of rate modifying constants

With the exception of the microbial biomass rate modifier (μ), all rate modifiers are based on established models (RothC and CENTURY) in order that differences between SOMic and traditional 1st order models can be attributed to the microbial dynamics rather than to different soil temperature and moisture responses.

1.4.1 Temperature

The rate modifying factor for temperature (τ) is given by a generalised Poisson function, which is the temperature response function of the CENTURY model[8] multiplied by a normalising coefficient (f_t) to give $\tau = 1.0$ at the average mean annual temperature of the calibration sites (10.8 °C).

$$\tau = f_t \left(\frac{T_{max} - T}{T_{max} - T_{opt}} \right)^{0.2} \mathrm{e}^{\frac{0.2}{2.63}\left(1 - \left(\frac{T_{max} - T}{T_{max} - T_{opt}}\right)^{2.63}\right)} \tag{3}$$

in which T is the soil temperature (°C), $f_t = 4.99$, $T_{max} = 45°\mathrm{C}$, and $T_{opt} = 35°\mathrm{C}$.

1.4.2 Moisture

The rate modifying factor for soil moisture (θ) is derived in one of two ways, depending on whether soil moisture data are available. If soil moisture is not known, as is often the case in field experiments (including the results presented in the main manuscript for long-term agricultural experiments), it is estimated from precipitation and potential evapotranspiration (PET) according to the RothC algorithm[1]:

$$\theta = \begin{cases} 1.0 & \text{if amd} > 0.444\text{max\_md}, \\ 0.2 + 0.8\frac{\text{max\_md} - \text{amd}}{0.556\text{max\_md}} & \text{if amd} \leq 0.444\text{max\_md}. \end{cases} \tag{4}$$

where max_md is the maximum possible soil moisture deficit, and amd is the accumulated moisture deficit (see ref. 1 for further detail on definitions and how these are calculated).

If soil moisture data are available, as for example in the calculation of global SOC stocks using soil moisture and temperature values from the Community Land Model (Section 2.4), the calculation of θ is based on a modified version of the RothC Equation, which uses the ratio of soil moisture to field capacity in the place of AMD:

$$\theta = \begin{cases} 1.0 & \text{if } \theta_m > 0.556\theta_f, \\ 0.2 + 0.8\frac{\theta_m}{0.556\theta_f} & \text{if } \theta_m \leq 0.556\theta_f, \end{cases} \tag{5}$$

where θ_m is the soil moisture and θ_f is the field capacity.

1.4.3 Soil cover

The soil cover rate modifying factor (ν) represents the faster mineralization of topsoil organic carbon following tillage that has removed vegetation. It is calculated according to the RothC method[1] as

$$\nu = \begin{cases} 0.6 & \text{if soil is vegetated}, \\ 1.0 & \text{if soil cover is bare due to tillage}. \end{cases} \tag{6}$$

1.4.4 Microbial biomass

SOC decomposition shows a non-linear response to enzyme concentration, which is believed to be due to competition for enzyme binding sites on substrates, as predicted by Langmuir adsorption isotherm theory[9]. This non-linear relationship can be approximated by a reverse Michaelis–Menten (MM) equation[2], which, assuming the approximation that exoenzyme production is proportional to microbial biomass[10], can be expressed as

$$\mu = \mu_{\max} \frac{[\mathrm{MB}]}{K_M + [\mathrm{MB}]} \tag{7}$$

where, μ is the rate modifying factor for microbial biomass, $\mu_{\max}$ is its maximum (enzyme saturated) value, [MB] is the concentration of microbial biomass, and the MM constant (K_M) is the [MB] at which the reaction rate is at half-maximum.

Whereas, in practice, different substrates could have different values of $\mu_{\max}$ and K_M, the simplifying assumption was made in SOMic version 1.0 to use common values for all microbially-mediated processes to keep the number of model parameters small and thus avoid overfitting.

1.5 Partitioning of DOC between sorption and microbial uptake

Only DOC is assumed to be taken up by microbes, because compounds must be in solution to cross the cell membrane. Microbes must also compete for DOC with abiotic sorption of DOC to minerals which also removes carbon from the DOC pool. The aggregate amount of C removed in a given time period from the DOC pool (D_{doc}) by both microbial uptake and sorption is given by

$$D_{doc} = \mathrm{DOC}_i \left(1 - e^{-k'_{doc}}\right), \tag{8}$$

where k'_{doc} is the modified rate constant for the combined processes of microbial uptake and sorption. Note that, although the form of equation 8 appears similar to a first-order reaction, the dependence of the rate factor k'_{doc} on reverse MM dynamics and on competition between microbes and sorption (see equations 9–13 below) ensures that it is not a simple 1st order process. k'_{doc} is calculated as the weighted mean of the modified rate constants for sorption (k'_{sorb}) and microbial uptake (k'_{mu}) according to

$$k'_{doc} = f_{sorb} k'_{sorb} + \left(1 - f_{sorb}\right) k'_{mu}, \tag{9}$$

in which the weighting applied to the sorption coefficient is the fraction (f_{sorb}) of D_{doc} that is sorbed, and the weighting for the microbial uptake is the remainder ($1 - f_{sorb}$) of D_{doc} that is taken up by microbes. The modified rate constant k'_{mu} is the product of the base rate constant for microbial uptake (k_{mu}) and the rate modifying factors itemised above in Section 1.4:

$$k'_{mu} = \tau \theta \nu \mu k_{mu}. \tag{10}$$

The modified rate constant k'_{sorb} is calculated similarly, but without any dependence on the microbial biomass rate-modifier (μ), instead having a rate modifying factor for soil clay content. For the clay rate modifier (c), we used a linear function of clay content (Equation 11), as is common practice in most current models (although future models may be improved by considering also clay mineralogy).[11,12]

$$c = 1 + m_{clay}\left(\mathrm{clay} - c_0\right), \tag{11}$$

where, m_c is the slope of the clay response function, and c_0 is the clay content at which c is equal to one. In order that c represents the change in rate constant relative to the average clay content in the calibration sites, c_0 was defined as the mean clay content at the calibration sites (23%). m_c was then calculated by numerical optimisation as one of the calibration parameters (see Section 1.8).

The modified rate constant k'_{sorb} is then given by

$$k'_{sorb} = \tau \theta \nu c k_{sorb}. \tag{12}$$

The fraction of carbon removed from the DOC pool due sorption can now be calculated from the modified sorption and microbial uptake rate constants as

$$f_{sorb} = \frac{k'_{sorb}}{k'_{sorb} + k'_{mu}} \tag{13}$$

whereby the carbon sorbed (S) during a time period is

$$S = D_{doc} f_{sorb}, \tag{14}$$

and the microbial uptake (M) is

$$\mathrm{M} = D_{doc}\left(1 - f_{sorb}\right). \tag{15}$$

1.6 Partitioning of microbial uptake between growth and respiration

Microbial uptake (M) is partitioned between growth (G) and respiration (R) by the microbial carbon use efficiency (CUE).

$$G = \mathrm{CUE}\ M \tag{16}$$

$$R = (1 - \mathrm{CUE})\ M \tag{17}$$

The balance between microbial growth and respiration, and thereby CUE, is known to vary with environmental parameters, and in particular temperature[13–17]. The temperature dependence of CUE was modelled as a linear function according to

$$\mathrm{CUE} = \mathrm{CUE}_0 - m_{cue}(T - 15), \tag{18}$$

where T is the soil temperature (°C), CUE_0 is the CUE at 15 °C, and m_{cue} is the rate of change of CUE with temperature. CUE_0 and m_{cue} were estimated by model calibration as described in Section 1.8 below.

1.7 Cycling of carbon between pools

Using the rate-modifying factors and partition functions derived above, the evolution of the MB, MAC, and DOC pools and respired CO_2–carbon over time is given by the following equations, in which the quantity of carbon in a pool in time period i is the carbon remaining from time $i-1$ plus the new carbon added to that pool:

$$\mathrm{MAC}_i = \mathrm{e}^{-\tau\theta\nu\mu k_{\mathrm{desorb}}}\mathrm{MAC}_{i-1} + f_{\mathrm{sorb}}\left(1-\mathrm{e}^{-k_{\mathrm{doc}}}\right)\mathrm{DOC}_{i-1} \tag{19}$$

$$\mathrm{MB}_i = \mathrm{e}^{-k_{\mathrm{mt}}}\mathrm{MB}_{i-1} + \mathrm{CUE}\left(1-f_{\mathrm{sorb}}\right)\left(1-\mathrm{e}^{-k_{\mathrm{doc}}}\right)\mathrm{DOC}_{i-1} \tag{20}$$

$$\mathrm{CO2}_i = \mathrm{CO2}_{i-1} + (1-\mathrm{CUE})\left(1-f_{\mathrm{sorb}}\right)\left(1-\mathrm{e}^{-k_{\mathrm{doc}}}\right)\mathrm{DOC}_{i-1} \tag{21}$$

$$\begin{aligned}\mathrm{DOC}_i = \mathrm{e}^{-k_{\mathrm{doc}}}\mathrm{DOC}_{i-1} &+ (1-\mathrm{e}^{-\tau\theta\nu\mu k_s})\mathrm{SPM}_{i-1} + (1-\mathrm{e}^{-\tau\theta\nu\mu k_i})\mathrm{IPM}_{i-1} + \\ &(1-\mathrm{e}^{-\tau\theta\nu\mu k_{\mathrm{mt}}})\mathrm{MB}_{i-1} + (1-\mathrm{e}^{-\tau\theta\nu\mu k_{\mathrm{desorb}}})\mathrm{MAC}_{i-1}\end{aligned} \tag{22}$$

where, k_{desorb} is the base rate constant for desorption of MAC, and k_{mt} is the base rate constant for microbial biomass turnover.

The dependence of MAC desorption rate on reverse MM dynamics in equation 19 is in accordance with observations that desorption of organic matter from mineral surfaces is accelerated by microbial enzyme activity, which in turn is limited by enzyme activity site availability.[18–21] Mechanisms by which microbial activity can destabilise and increase desorption of MAC include, for example, the destabilisation of soil aggregates by decomposition of organic binding agents[22]; the utilisation of sorbed organic compounds by microorganisms that adhere to the mineral surfaces[22–24]; microbial transformation of sorbed organic compounds into more readily desorbed compounds[25]; and displacement by microbial exudates[21].

1.7.1 *Differential form of model equations*

The rate of change of carbon in each pool can also be written in differential form (equations 23–27 below) to make it easier to see structure of the carbon fluxes between pools. For brevity, the model carbon pools in Eq. 23–27 are denoted as C_1 (SPM), C_2 (IPM), C_3 (DOC), C_4 (MAC), and C_5 (MB). Note that, in these differential equations, k'_1, k'_2, k'_3, k'_4, and k'_5 are the decomposition rate factors for the pools C_1 to C_5, respectively, *after* the rate modifying factors have been applied. Therefore, although these differential equations appear similar in form to first order decay, the dependence of the rate factors on the size of other pools ensures that they are not, in fact, first order. In particular, the dependence of k'_1, k'_2, k'_3, k'_4, and k'_5 on microbial biomass abundance through MM means that these factors are not constants but are themselves dynamic quantities.

$$\frac{\mathrm{d}C_1}{\mathrm{d}t} = f_s\frac{\mathrm{d}L}{\mathrm{d}t} - k'_1C_1 \tag{23}$$

$$\frac{\mathrm{d}C_2}{\mathrm{d}t} = (1-f_s)\frac{\mathrm{d}L}{\mathrm{d}t} - k'_2C_2 \tag{24}$$

$$\frac{\mathrm{d}C_3}{\mathrm{d}t} = k'_1C_1 + k'_2C_2 + k'_4C_4 + k'_5C_5 - k'_3C_3 \tag{25}$$

$$\frac{\mathrm{d}C_4}{\mathrm{d}t} = f_{\mathrm{sorb}}k'_3C_3 - k'_4C_4 \tag{26}$$

$$\frac{\mathrm{d}C_5}{\mathrm{d}t} = \mathrm{CUE}(1-f_{\mathrm{sorb}})k'_3C_3 - k'_5C_5 \tag{27}$$

$$\frac{\mathrm{dCO_2}}{\mathrm{d}t} = (1-\mathrm{CUE})(1-f_{\mathrm{sorb}})k'_3C_3, \tag{28}$$

where, $\frac{\mathrm{dCO_2}}{\mathrm{d}t}$ is the rate of respired CO_2 evolution; and $\frac{\mathrm{d}L}{\mathrm{d}t}$ is the rate of fresh plant litter input to the soil.

1.8 Calibration of the model

Calibration of the model was accomplished by minimising the sum of squares of the residuals for the calibration data using the quasi-Newton Broyden–Fletcher–Goldfarb–Shanno (BFGS) method[26]. The model parameters that were optimised, and their calibrated values, are given in Table 1.

2 Data sources

2.1 Pendleton long-term residue management experiment

The long-term residue management experiment at Pendleton, Oregon USA is maintained by the Columbia Basin Agricultural Research Center of Oregon State University, located at 45°44' N, 118°37' W. The soil is a coarse silty mixed mesic Typic Haploxeroll (USDA classification), with 18% clay[27]. Crop rotation is a 2-year winter wheat/fallow system, with a 15–month fallow and a 9–month cropping season. The climate is semi-arid. Temperature, precipitation, and PET were calculated using the Food and Agriculture Organisation of the United Nations' (FAO's) New_LocClim climate-station database and interpolation software, by inverse distance weighted average (IDWA), with vertical and horizontal regression correction, from a maximum of 50 climate stations within a maximum of 1000 km (Table 2).

The nine treatments modeled here varied in terms of mineral nitrogen fertilizer additions, residue management (fall burning, spring burning, or no burning), and organic additions (none, manure, or pea vine), as described in Table 3.

Above-ground carbon additions to the soil are from Rasmussen and Parton[28]. The experiment was revised in 1967 to change the wheat type from a medium-tall to a semi-dwarf variety. Below-ground carbon allocation was estimated using a root:shoot ratio of 0.625 for the long-straw variety, and 0.5325 for the short straw[29]. This provided the estimated total carbon inputs in Table 4.

Soil carbon measurements from Rasmussen et al.[27] are given in Table 5.

2.2 Sanborn Field

Sanborn Field, is located on the campus of the University of Missouri-Columbia (39.03° N, 94.58° W), and was established in 1888. The soil is a Mexico silt loam with 28.3% mean clay content in the maize and wheat plots modelled[30]. Mean annual temperature is 13 °C, mean annual precipitation is 973 mm, and mean annual potential evapotranspiration is 790 mm[31]. Monthly mean values of these climate parameters are given in Table 6.

The experiment station comprises rotation and manure treatments on 39 plots. The treatments modelled were continuous maize with full mineral fertiliser and regular tillage (cmf), continuous maize with no fertiliser of manure (cmn), continuous wheat with full mineral fertiliser (cwf), continuous wheat with manure (cwm), and continuous wheat with no fertiliser of manure (cwn). Total carbon additions to the soil from crop residues and below ground production are given in Table 3 of Buyanovsky and Wagner[31]. In addition, the manure treatments received 13.4 Mg manure annually. The Phyllis2 Database for biomass and waste[32] gives mean water content of farmyard manure as 70%, mean ash content 33 %, and mean dry ash-free carbon content as 48 %. On this basis, the annual carbon addition in manure was estimated at 1.3 Mg C y^{-1}. Details on the management of the treatments and soil carbon measurements can be found in Buyanovsky and Wagner[31].

2.3 Rothamsted Experimental Station

The Rothamsted Experimental Farm (51°49' N, 0°21' W) is the oldest long-term agricultural experiment site globally, established in 1843. The soil is a silty clay loam (FAO classification is Chromic Luvisol) with an average 23.4% clay content[33]. Complete experimental data including annual management, yields, meteorology and soil carbon are available from the Rothamsted electronic archive[34], with a summary of the modelled sites provided here. The mean annual temperature is 9.2 °C, mean annual precipitation is 704 mm, and mean annual potential evapotranspiration is 450 mm[1]. Monthly mean values are given in Table 7.

Broadbalk: The first experimental winter wheat crop was sown on Broadbalk in autumn 1843, and every year since then[35]. All plots received annual returns of dead crop material (roots, stubble and part of the chaff), and were fallow only between Aug harvest and Nov sowing. Treatments varied according to the amounts and types of mineral and organic fertilisers that were applied (Table 8). Wheat straw was assumed to be 85% dry matter[34], and 48.79% C[32]. In the plots that received 35 Mg ha^{-1} y^{-1} farmyard manure, this was assumed to provide 3 Mg C ha^{-1} y^{-1}[33].

Hoosfield: The Hoosfield experiment was started in 1852. Spring barley grown every year except 1912, 1933, 1943 and 1967 when the whole experiment was fallowed to control weeds. In contrast to Broadbalk, because it is spring sown, it has only been necessary to fallow these four times to control weeds[34]. The annual input of plant residues from the barley was estimated as 2.80 Mg C ha^{-1} y^{-1} in treatments and years in which farmyard manure was applied, and 1.6 Mg C ha^{-1} y^{-1} in unmanured treatments and years[1]. The modelled treatments are described in Table 9. In the plots that received 35 Mg ha^{-1} y^{-1} farmyard manure, this was assumed to provide 3 Mg C ha^{-1} y^{-1}[33].

2.4 Global SOC distribution

For the estimate of global SOC stocks and distribution, Community Land Model (CLM) spatial output data were used to estimate soil temperature, moisture and litter inputs. CLM version 4.5CN data were from the historic land post-processed monthly-average data for the years 1850–2010[36] available on request from the National Center for Atmospheric Research (NCAR) at `https://www.earthsystemgrid.org`.

Gridded monthly litter-fall was obtained from the CLM model output file `clm45cn_1deg4502_hist.clm2.h0.LITFALL.185001-201012.nc`. Gridded monthly litter heterotrophic respiration was from the CLM model output file `clm45cn_1deg4502_hist.clm2.h0.LITHR.185001-201012.nc`. Litter input to the mineral soil was calculated as litter-fall minus litter heterotrophic respiration.

Soil temperature in the top 0.3 m was calculated as the weighted mean of the top 5 layers from the CLM output file `clm45cn_1deg4502_hist.clm2.h0.TSOI.185001-201012.nc` (weighted by layer thickness). Soil moisture was the weighted mean of the top five layers of `clm45cn_1deg4502_hist.clm2.h0.H2OSOI.185001-201012.nc`. Saturation capacity (θ_s; m^3m^{-3}) was estimated using the following equation

$$\theta_s = 0.14\,\text{clay} - 0.34 D_b + 0.86, \tag{29}$$

where, D_b is bulk density (Mg m^{-3}), and clay is in percent[37].

Global gridded bulk density and soil texture estimates were obtained from the regridded harmonized world soils database v 1.2[38].

Global ecoregions were based on the classification from Olson and Dinerstein[39], with the associated spatial data available at `http://maps.tnc.org/files/shp/terr-ecoregions-TNC.zip`.

3 Radiocarbon age of SOC

The age of SOC as a function of depth was calculated by running the model on a sequence of soil layers from the top horizon down to below 1 m depth, with DOC leaching represented by the quantity of DOC removed from each soil layer in each time step being added to the next lower layer. Because C is exchanged between DOC, MAC, and MB as it percolates through the soil column, the mean DOC age in a soil layer is always (i) younger than the mean SOC age in that layer (because it contains some younger DOC added from fresh litter or from the layer above), and (ii) older than the fresh litter or DOC inputs, because it contains some C derived from desorbed MAC or MB turnover. This means that the DOC input into subsequent layers increases incrementally in age (the mean age of DOC in a layer is always older than the fresh C inputs into that layer), in a process that has been described as ”cycling downwards”[25].

The soil layer thicknesses were based on the CLM depth definitions and are given in Table 10. In each time period, the exchange of C between the DOC, MAC, and MB pools is first calculated according to the Equation 22. Then DOC advection from one layer to the next was calculated using an advection velocity estimated using CLM values for groundwater recharge rate for the years 1850–2010[36]. Time averaged water velocity for the Rothamsted site varies with depth from a mean surface infiltration rate of 310 mm yr^{-1} to an aquifer recharge rate of 45mm yr^{-1}—a value that averages saturated and unsaturated flow conditions over time. Daily values can be obtained from the CLM model output file `clm45cn_1deg4502_hist.clm2.h0.QCHARGE.185001-201012.nc` available on request from the National Center for Atmospheric Research (NCAR). Soil temperature and moisture in each layer were also taken from the same CLM run, in files `clm45cn_1deg4502_hist.clm2.h0.TSOI.185001-201012.nc` and `clm45cn_1deg4502_hist.clm2.h0.H2OSOI.185001-201012.nc`, respectively. To estimate the radiocarbon age profile at a specific location, the vertically layered model with DOC advection was spun up over 12,000 years using the litter input values and gridded soil characteristics as described in Section 2.4. For example, using the CLM forcing data at Rothamsted (51°49’ N, 0°21’ W) give predicted versus measured[40] radiocarbon ages with an adjusted $R^2 = 0.67$, $p < 2.2x10\text{-}16$, n=78) (Fig. 1). Note that the 78 data points from ref. 40 excluded measurements from the Park Grass surface soils, which had anomalously old ^{14}C ages, due to contamination with coal.

4 Supplementary Tables

Table 1. Model parameters, and their calibrated values.

Parameter	Description	Value	Unit
μ_{max}	the Michaelis–Menten saturation coefficient	1.11	dimensionless
K_m	the Michaelis–Menten half-maximum coefficient	0.409	Mg ha^{-1}
k_s	the base rate constant for SPM dissolution	9.47x10^{-7}	s^{-1}
k_i	the base rate constant for IPM depolymerization	1.66x10^{-8}	s^{-1}
k_{mu}	the base rate constant for microbial uptake	5.52x10^{-7}	s^{-1}
k_{mt}	the base rate constant for microbial turnover	8.97x10^{-8}	s^{-1}
k_{sorb}	the base rate constant for sorption	1.17x10^{-7}	s^{-1}
k_{desorb}	the base rate constant for desorption	2.09x10^{-9}	s^{-1}
CUE_0	the microbial carbon use efficiency at 15 °C	0.281	dimensionless
m_{cue}	the temperature dependence of carbon use efficiency	0.0081	°C^{-1}
m_{clay}	the clay dependence coefficient of sorption/desorption	0.0145	%clay^{-1}

Table 2. Mean monthly temperature (T), precipitation (PREC), and potential evapotranspiration (PET) at Pendleton OR.

	Jan	Feb	Mar	Apr	May	Jun	Jul	Aug	Sep	Oct	Nov	Dec
T (°C)	-0.18	2.85	5.93	8.97	12.93	17.16	20.85	20.43	15.56	9.82	4.46	0.42
PREC (mm)	48.73	36.14	41.33	35.44	34.74	25.91	11.02	17.38	20.83	30.10	51.84	51.92
PET (mm)	17.97	28.99	56.77	85.73	123.51	150.19	182.78	163.25	109.71	66.11	30.07	17.28

Table 3. Mineral fertilizer (kg N ha^{-1} crop^{-1}) and organic additions (Mg wet organic matter ha^{-1} crop^{-1}), and residue management in the treatments at Pendleton OR. sB = spring burn, fB = fall burn, nB = not burned, fD = fall disking, sD = spring disking.

	Residue Management			Organic Addition			Mineral N fertilizer		
Treatment	1931–1966	1967–1978	1979–1986	1931–1966	1967–1978	1979–1986	1931–1966	1967–1978	1979–1986
fB_N0	fB	fB	fB	0	0	0	0	0	0
sB_N0	sB	sB	sB	0	0	0	0	0	0
nB_N0	nB	nB	nB	0	0	0	0	0	0
sB_N45	fD	nB	sB	0	0	0	0	45	45
sB_N90	sD	nB	sB	0	0	0	0	90	90
nB_N45	fD	nB	nB	0	0	0	34	45	45
nB_N90	sD	nB	nB	0	0	0	34	90	90
nB_PV	nB	nB	nB	2.24	2.24	2.24	0	0	0
nB_MN	nB	nB	nB	22.40	22.40	22.40	0	0	0

Table 4. Total organic carbon inputs to the soil at Pendleton OR, including above-ground residues, below-ground net primary production, and organic amendments (Mg C ha^{-1} crop^{-1}).

Treatment	1931–1941	1942–1951	1952–1966	1967–1976	1977–1986
fB_N0	0.94	0.94	0.73	1.04	1.06
sB_N0	1.58	1.64	1.33	1.76	1.82
nB_N0	1.82	1.93	1.60	2.10	2.19
sB_N45	3.25	3.20	2.73	2.05	2.42
sB_N90	3.29	3.36	2.69	2.16	2.62
nB_N45	2.18	2.34	2.12	2.53	2.95
nB_N90	2.13	2.32	2.07	2.68	3.13
nB_PV	2.267	2.477	2.177	2.117	2.247
nB_MN	2.688	2.298	2.818	3.318	3.318

Table 5. Soil organic carbon (0–0.3 m) at Pendleton OR (Mg C ha^{-1})[27].

Treatment	1931	1941	1951	1964	1976	1986
fB_N0	48.67	46.44	42.15	42.45	40.01	37.01
sB_N0	48.12	45.74	42.69	43.18	41.42	38.96
nB_N0	50.21	49.23	44.93	44.00	42.47	39.65
sB_N45	49.24	48.08	43.78	42.72	41.31	38.74
sB_N90	49.41	47.08	42.8 0	41.81	41.52	39.95
nB_N45	49.90	48.36	45.96	44.91	44.24	41.36
nB_N90	48.75	48.59	46.18	44.09	43.65	41.90
nB_PV	49.92	50.48	50.63	47.01	46.15	44.49
nB_MN	48.57	50.88	53.07	49.39	50.8	50.18

Table 6. Mean monthly temperature (T), precipitation (PREC), and potential evapotranspiration (PET) at Sanborn Field, MO.

	Jan	Feb	Mar	Apr	May	Jun	Jul	Aug	Sep	Oct	Nov	Dec
T (°C)	-1.5	1.1	7	13.5	18.5	23	26	24.8	20.6	14.5	7.5	0.8
PREC (mm)	36	45.8	79	95.5	124.9	107.6	91.4	81.8	96.2	80.3	73	61.5
PET (mm)	22.3	24.4	47.1	73.8	87.7	102.4	120.2	107.7	81.1	63.3	37.3	22.7

Table 7. Mean monthly temperature (T), precipitation (PREC), and potential evapotranspiration (PET) at Rothamsted, UK.

	Jan	Feb	Mar	Apr	May	Jun	Jul	Aug	Sep	Oct	Nov	Dec
T (°C)	3.4	3.6	5.1	7.3	11	13.9	16	16	13.5	10.2	6.1	4.6
PREC (mm)	74	59	62	51	52	57	34	55	58	56	75	71
PET (mm)	6	7.5	20.2	36.8	62.2	74.2	77.2	68.2	51.8	25.5	13.5	6

Table 8. Description of modeled treatment plots in the Broadbalk long-term continuous winter wheat experiment at Rothamsted, UK.

Plot	Treatment
bb3	No fertilizer or organic amendments.
bb5	Mineral fertilizer: 35 kg P ha^{-1}, 90 kg K ha^{-1}, Na, and Mg. No organic amendments, except that straw was chopped and returned to the plots for 11 years (1869-1879).
bb8	Mineral fertilizer: 144 kg N ha^{-1}, 35 kg P ha^{-1}, 90 kg K ha^{-1}, Na, and Mg. No organic amendments, except that straw was chopped and returned to the plots for 11 years (1869-1879).
bb21	35 Mg ha^{-1} fresh farmyard manure every year since 1885
bb22	35 Mg ha^{-1} fresh farmyard manure every year since 1843

Table 9. Description of modeled treatment plots in the Hoosfield long-term continuous winter wheat experiment at Rothamsted, UK.

Plot	Treatment
hoos6	No fertilizer or organic amendments.
hoos7_1	35 Mg ha^{-1} y^{-1} farmyard manure, 1852-1871 only.
hoos7_2	35 Mg ha^{-1} y^{-1} farmyard manure.

Table 10. Boundary depths for soil layers (up to 1 m) defined in the community land model.

Layer	Lower boundary depth (cm)
1	1.8
2	4.5
3	9.1
4	16.6
5	28.9
6	49.3
7	82.9
8	138.3
9	229.6
10	380.2
11	628.4
12	1037.8

5 Supplementary Figures

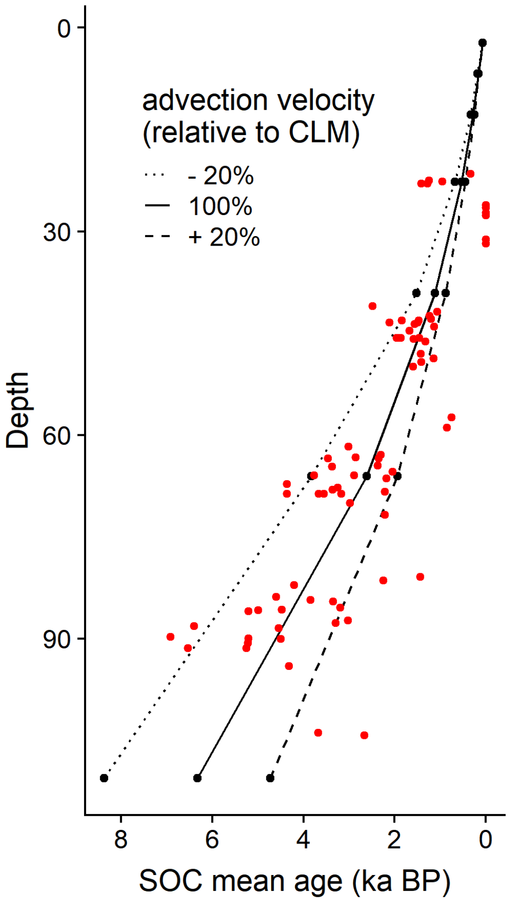

Figure 1. Mean age of SOC (in thousands of years before present; ka BP) as a function of depth at Rothamsted, UK. Red dots are measured values, and the black lines indicates the SOMic model prediction. The solid black line shows predicted values when forced witrh CLM water transport estimates. The dotted and dashed black lines show a sensitivity of the prediction to a change in advection velocity of +/- 20% relative to the CLM values.

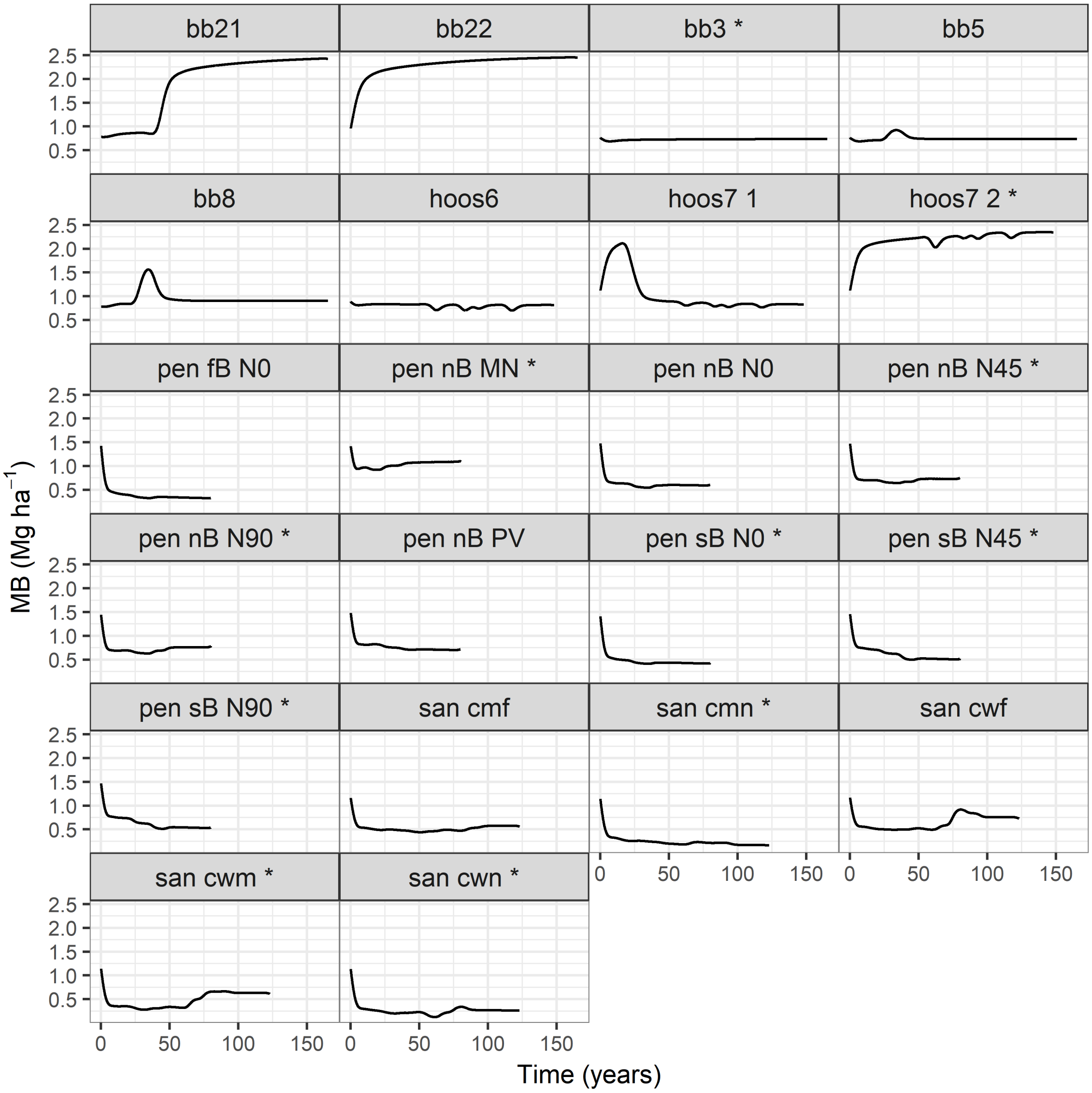

Figure 2. Microbial biomass (MB) stocks in the top soil horizon of twenty-two long-term agricultural experiments in Rothamsted, UK ("bb", and "hoos" treatments), Pendleton OR, USA ("Pen"), and Sanborn MO, USA ("San"), as predicted using the SOMic 1.0 model.

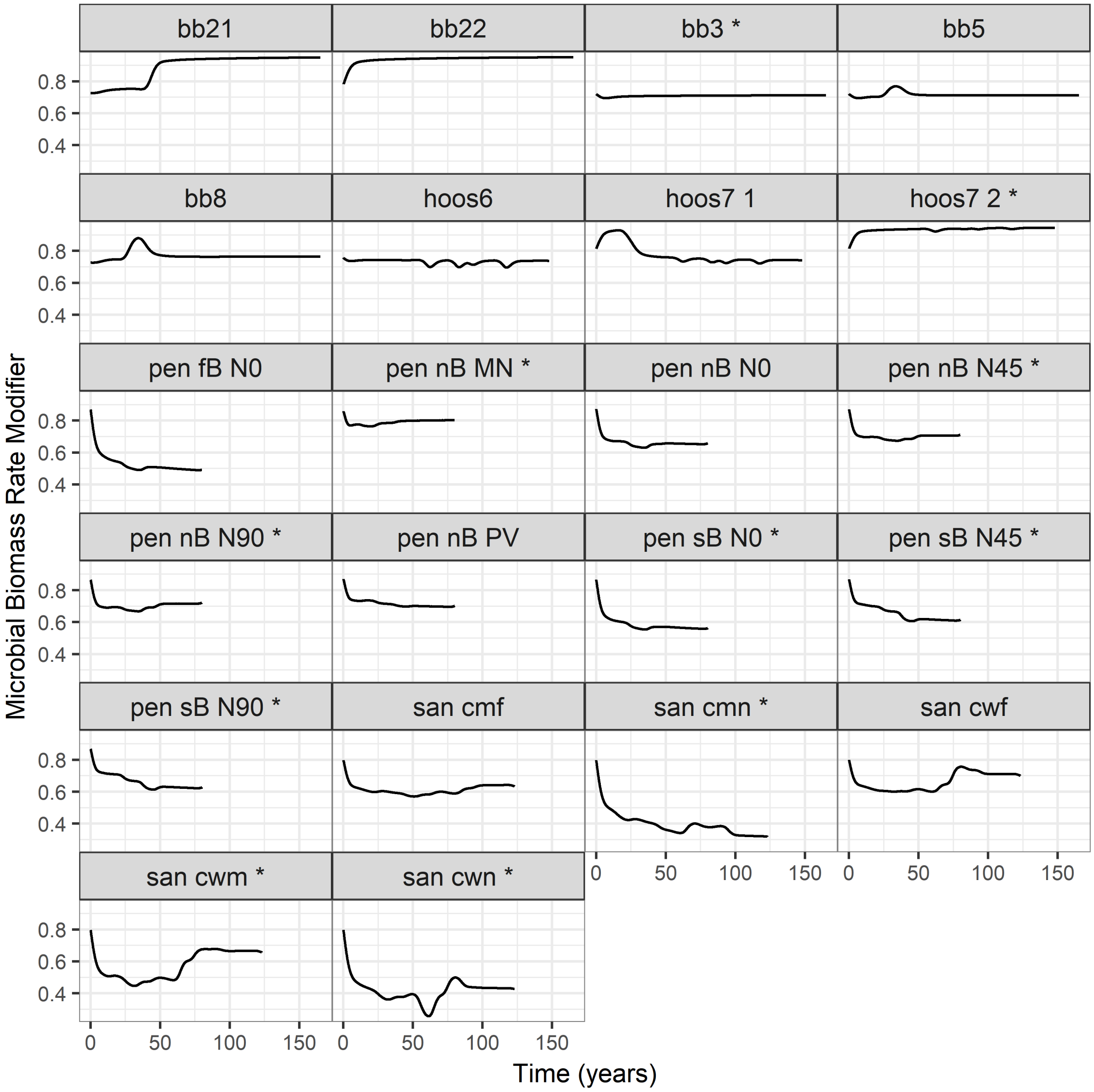

Figure 3. Rate modifying factor for microbial biomass (μ) in the top soil horizon of twenty-two long-term agricultural experiments in Rothamsted, UK ("bb", and "hoos" treatments), Pendleton OR, USA (”Pen”), and Sanborn MO, USA (”San”), as predicted using the SOMic 1.0 model.

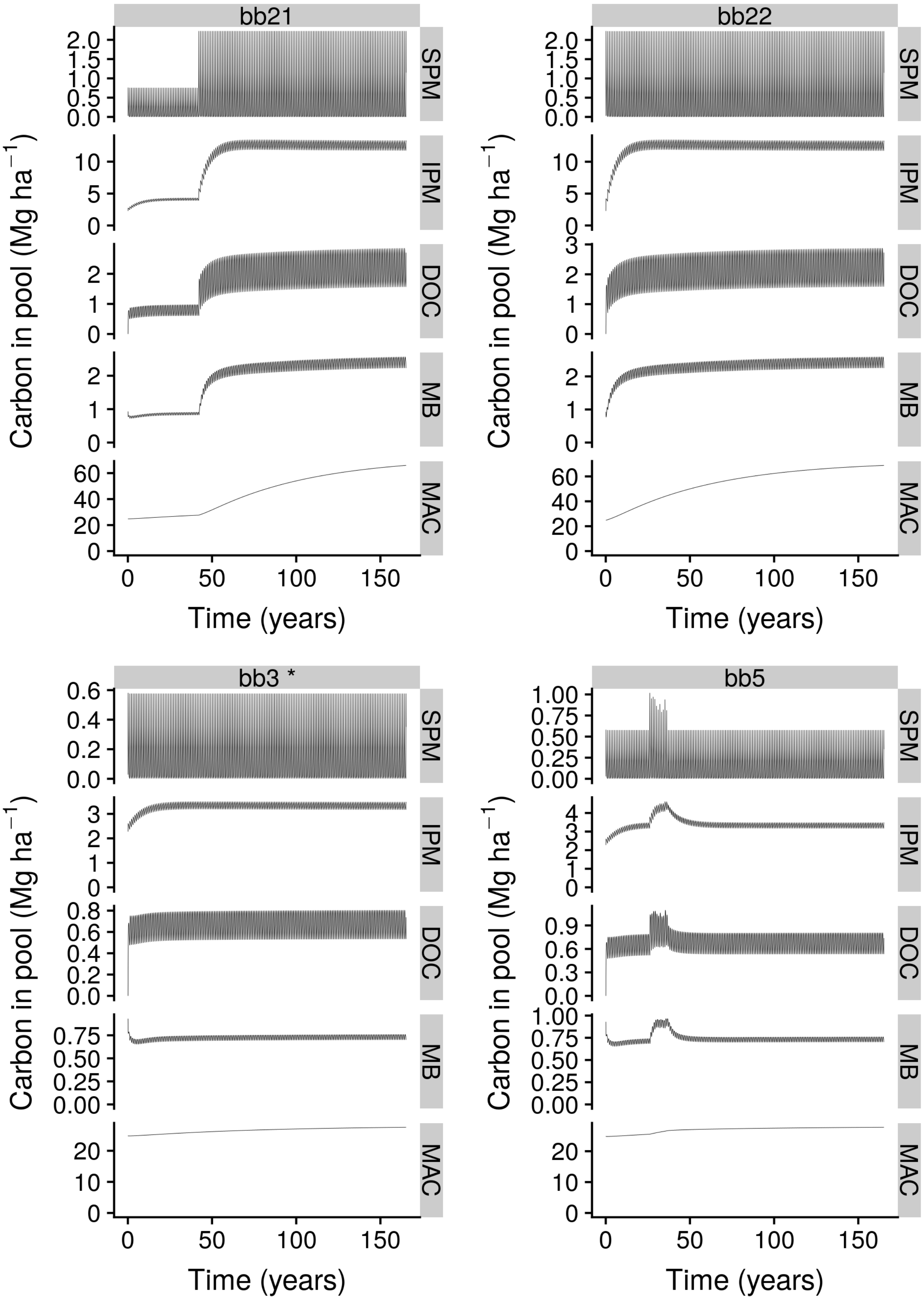

Figure 4. Soil organic carbon as a function of time in the five pools of the SOMic 1.0 model, at the sites bb21, bb22, bb3 and bb5.

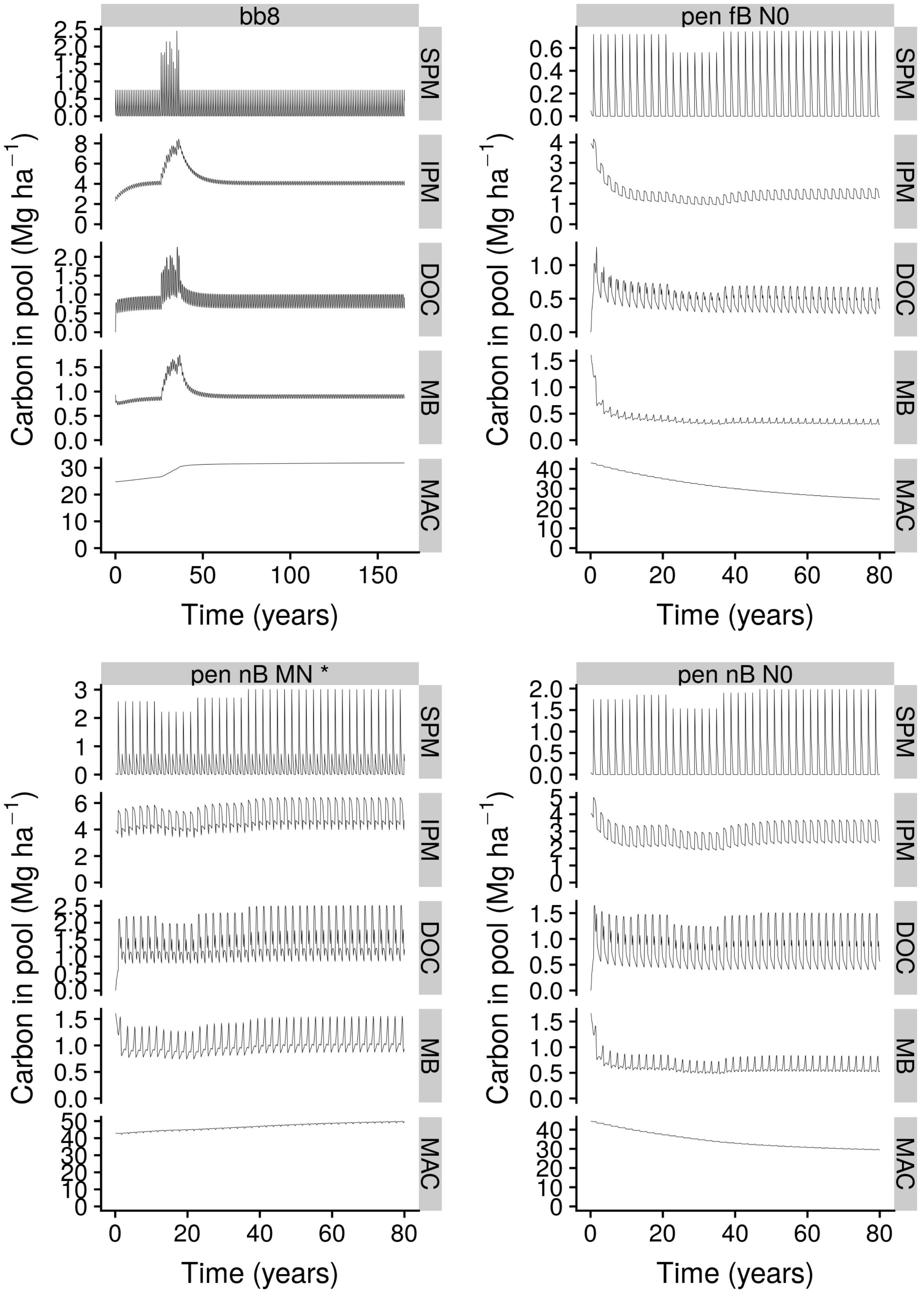

Figure 5. Soil organic carbon as a function of time in the five pools of the SOMic 1.0 model, at the sites bb8, pen fB N0, pen nB MN and pen nB N0.

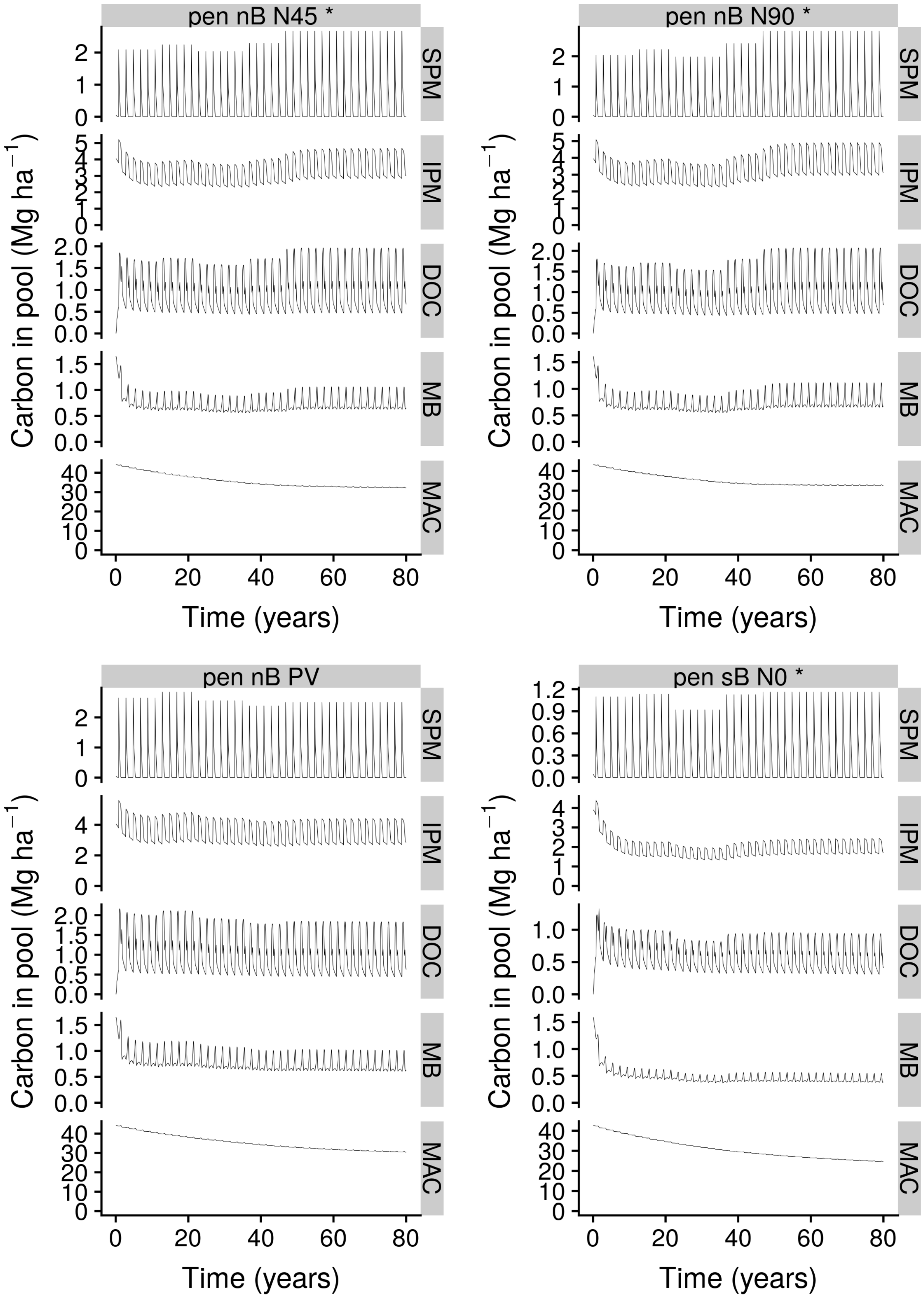

Figure 6. Soil organic carbon as a function of time in the five pools of the SOMic 1.0 model, at the sites pen nB N45, pen nB N90 and pen nB PV and pen sB N0.

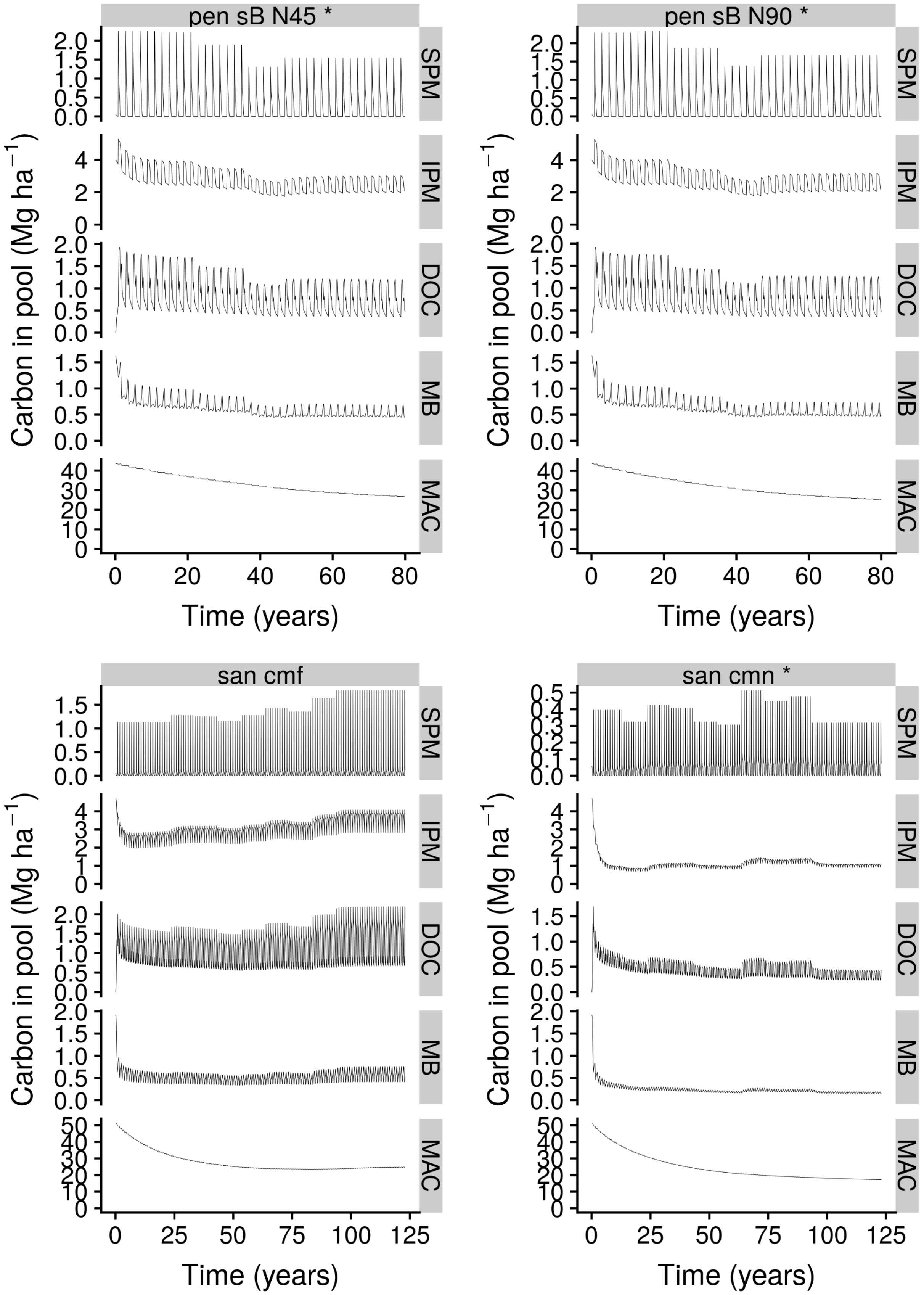

Figure 7. Soil organic carbon as a function of time in the five pools of the SOMic 1.0 model, at the sites pen sB N45, pen sB N90 and san cmf, and san cmn.

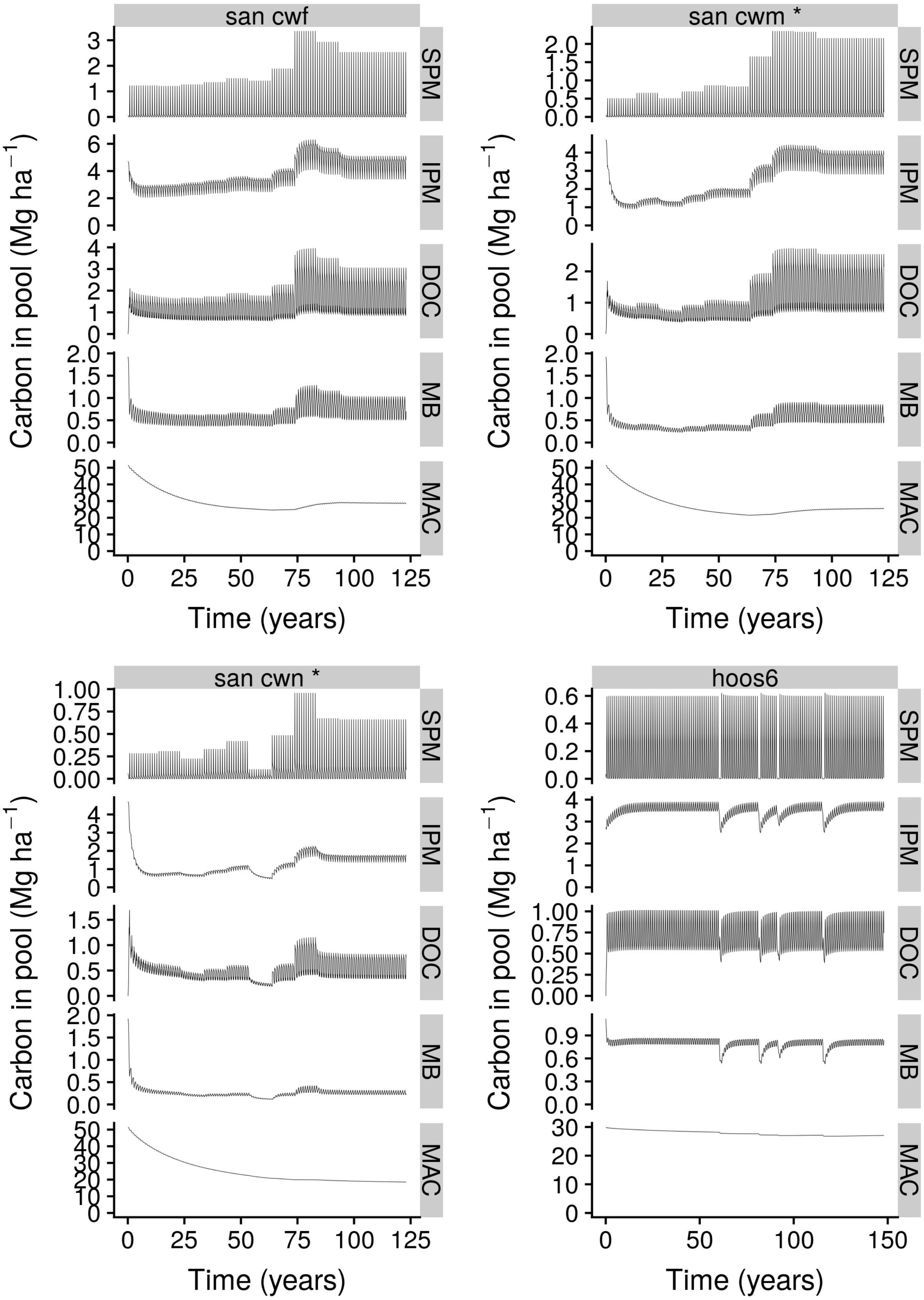

Figure 8. Soil organic carbon as a function of time in the five pools of the SOMic 1.0 model, at the sites san cwf, san cwm, san cwn, and hoos 6.

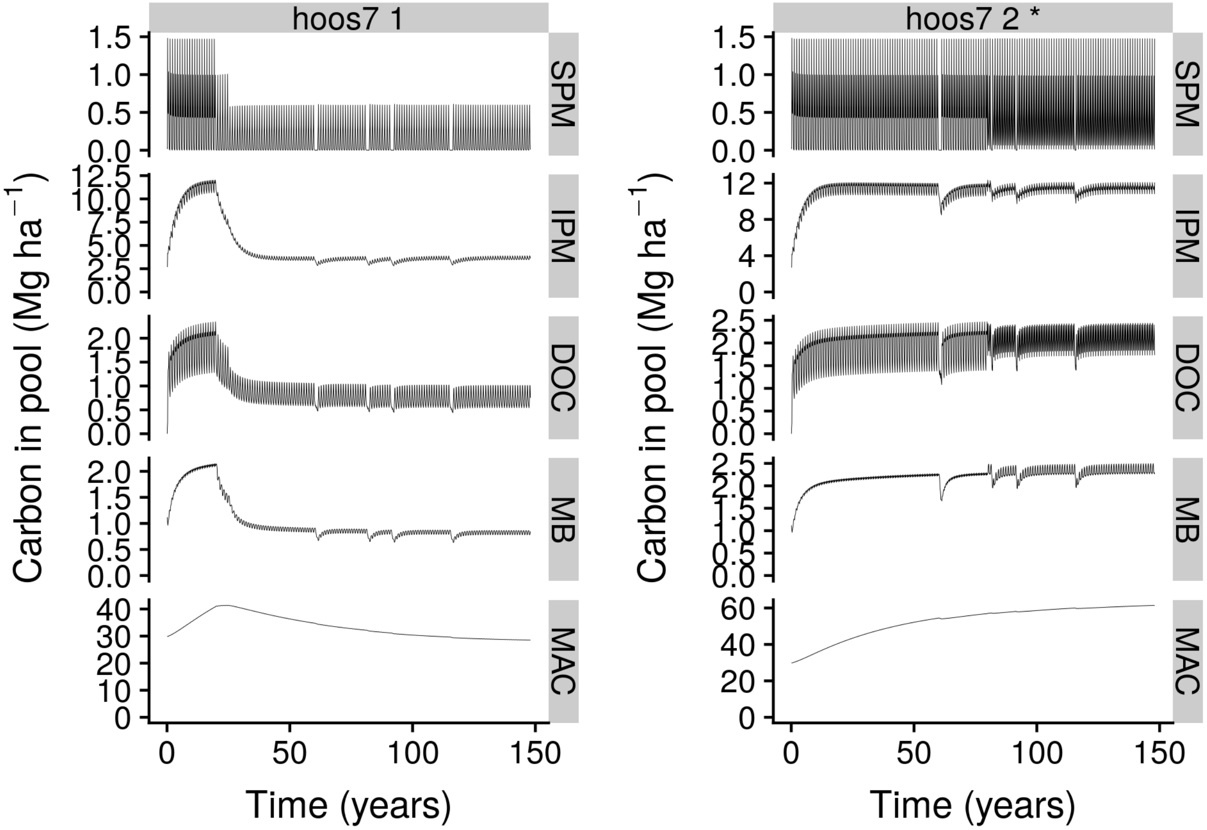

Figure 9. Soil organic carbon as a function of time in the five pools of the SOMic 1.0 model, at the sites hoos 7_1 and hoos 7_2.

References

1. Coleman, K. & Jenkinson, D. *ROTHC-26.3* (Rothamsted Research, Harpenden, Herts, UK, 2008).
2. Schimel, J. P. & Weintraub, M. N. The implications of exoenzyme activity on microbial carbon and nitrogen limitation in soil: a theoretical model. *Soil Biol. Biochem.* **35**, 549–563, DOI: 10.1016/S0038-0717(03)00015-4 (2003).
3. Guggenberger, G., Zech, W. & Schulten, H.-R. Formation and mobilization pathways of dissolved organic matter: evidence from chemical structural studies of organic matter fractions in acid forest floor solutions. *Org. Geochem.* **21**, 51–66 (1994).
4. Møller, J., Miller, M. & Kjøller, A. Fungal–bacterial interaction on beech leaves: influence on decomposition and dissolved organic carbon quality. *Soil Biol. Biochem.* **31**, 367–374 (1999).
5. Don, A. & Kalbitz, K. Amounts and degradability of dissolved organic carbon from foliar litter at different decomposition stages. *Soil Biol. Biochem.* **37**, 2171–2179 (2005).
6. Hagedorn, F. & Machwitz, M. Controls on dissolved organic matter leaching from forest litter grown under elevated atmospheric co2. *Soil Biol. Biochem.* **39**, 1759–1769 (2007).
7. Kalbitz, K. *et al.* Changes in properties of soil-derived dissolved organic matter induced by biodegradation. *Soil Biol. Biochem.* **35**, 1129–1142 (2003).
8. Burke, I. C. *et al.* Evaluating and testing models of terrestrial biogeochemistry: the role of temperature in controlling decomposition. *Model. ecosystem science. Princet. Univ. Press. Princeton, New Jersey, USA* 225–253 (2003).
9. Vetter, Y. A., Deming, J. W., Jumars, P. A. & Krieger-Brockett, B. B. A Predictive Model of Bacterial Foraging by Means of Freely Released Extracellular Enzymes. *Microb. Ecol.* **36**, 75–92, DOI: 10.1007/s002489900095 (1998).
10. Todd-Brown, K. E. O., Hopkins, F. M., Kivlin, S. N., Talbot, J. M. & Allison, S. D. A framework for representing microbial decomposition in coupled climate models. *Biogeochemistry* **109**, 19–33, DOI: 10.1007/s10533-011-9635-6 (2012).
11. Rasmussen, C. *et al.* Beyond clay: towards an improved set of variables for predicting soil organic matter content. *Biogeochemistry* 1–10, DOI: 10.1007/s10533-018-0424-3 (2018).
12. Kaiser, K. & Guggenberger, G. Mineral surfaces and soil organic matter. *Eur. J. Soil Sci.* **54**, 219–236 (2003).
13. Allison, S. D., Wallenstein, M. D. & Bradford, M. A. Soil-carbon response to warming dependent on microbial physiology. *Nat. Geosci.* **3**, 336 (2010).
14. Manzoni, S. & Porporato, A. Soil carbon and nitrogen mineralization: Theory and models across scales. *Soil Biol. Biochem.* **41**, 1355–1379, DOI: 10.1016/j.soilbio.2009.02.031 (2009).
15. Li, J., Wang, G., Allison, S. D., Mayes, M. A. & Luo, Y. Soil carbon sensitivity to temperature and carbon use efficiency compared across microbial-ecosystem models of varying complexity. *Biogeochemistry* **119**, 67–84, DOI: 10.1007/s10533-013-9948-8 (2014).
16. Sinsabaugh, R. L., Manzoni, S., Moorhead, D. L. & Richter, A. Carbon use efficiency of microbial communities: stoichiometry, methodology and modelling. *Ecol. Lett.* **16**, 930–939, DOI: 10.1111/ele.12113 (2013).
17. Sinsabaugh, R. L. *et al.* Stoichiometry of microbial carbon use efficiency in soils. *Ecol. Monogr.* **86**, 172–189, DOI: 10.1890/15-2110.1 (2016).
18. Guggenberger, G. & Kaiser, K. Dissolved organic matter in soil: challenging the paradigm of sorptive preservation. *Geoderma* **113**, 293–310 (2003).
19. McGhee, I., Sannino, F., Gianfreda, L. & Burns, R. G. Bioavailability of 2, 4-d sorbed to a chlorite-like complex. *Chemosphere* **39**, 285–291 (1999).
20. Singh, N. *et al.* Bioavailability of an organophosphorus pesticide, fenamiphos, sorbed on an organo clay. *J. agricultural food chemistry* **51**, 2653–2658 (2003).
21. Mikutta, R. *et al.* Biodegradation of forest floor organic matter bound to minerals via different binding mechanisms. *Geochimica et Cosmochimica Acta* **71**, 2569–2590 (2007).
22. Huang, P.-M., Wang, M.-K. & Chiu, C.-Y. Soil mineral–organic matter–microbe interactions: impacts on biogeochemical processes and biodiversity in soils. *Pedobiologia* **49**, 609–635 (2005).
23. Alexander, M. *Biodegradation and bioremediation* (Gulf Professional Publishing, 1999).
24. Digrazia, P. M. *et al.* Development of a systems analysis approach for resolving the structure of biodegrading soil systems. *Appl. Biochem. Biotechnol.* **24**, 237–252 (1990).

25. Kaiser, K. & Kalbitz, K. Cycling downwards–dissolved organic matter in soils. *Soil Biol. Biochem.* **52**, 29–32 (2012).

26. Shanno, D. F. Conditioning of quasi-Newton methods for function minimization. *Math. computation* **24**, 647–656 (1970).

27. Rasmussen, P., Smiley, R. & Albrecht, S. Long-term residue management experiment: Pendleton, oregon usa. In *Evaluation of Soil Organic Matter Models*, 391–396 (Springer, 1996).

28. Rasmussen, P. E. & Parton, W. J. Long-term effects of residue management in wheat-fallow: I. Inputs, yield, and soil organic matter. *Soil Sci. Soc. Am. J.* **58**, 523–530 (1994).

29. Siddique, K. H. M., Belford, R. K. & Tennant, D. Root:shoot ratios of old and modern, tall and semi-dwarf wheats in a mediterranean environment. *Plant Soil* **121**, 89–98, DOI: 10.1007/BF00013101 (1990).

30. Gantzer, C. J., Anderson, S. H., Thompson, A. L. & Brown, J. R. Estimating soil erosion after 100 years of cropping on Sanborn Field. *J. Soil Water Conserv.* **45**, 641–644 (1990).

31. Buyanovsky, G. A. & Wagner, G. H. Carbon cycling in cultivated land and its global significance. *Glob. Chang. Biol.* **4**, 131–141, DOI: 10.1046/j.1365-2486.1998.00130.x (1998).

32. ECN. Phyllis2 - Database for biomass and waste (2015).

33. Jenkinson, D., Harkness, D., Vance, E., Adams, D. & Harrison, A. Calculating net primary production and annual input of organic matter to soil from the amount and radiocarbon content of soil organic matter. *Soil Biol. Biochem.* **24**, 295–308 (1992).

34. BBSRC (Biotechnology and Biological Sciences Research Council). http://www.era.rothamsted.ac.uk. Accessed: 2017-04-30.

35. Poulton, P. Rothamsted research: guide to the classical and other long-term experiments, datasets and sample archive. *Rothamsted Res. Harpenden, UK* (2006).

36. National Center for Atmospheric Research (NCAR). https://www.earthsystemgrid.org/dataset/ucar.cgd.ccsm4.CLM4.5CN.lnd.proc.monthly_ave.html. Accessed: Aug 2017.

37. Scheinost, A. Pedotransfer-funktionen zum wasser-und stoffhaushalt einer bodenlandschaft.(pedo-transfer functions for water and matter balances of a soilscape. in german, with english abstract.) phd thesis. *Tech. Univ. München, Freising-Weihenstephan, Ger.* (1995).

38. Wieder, W., Boehnert, J., Bonan, G. & Langseth, M. Regridded Harmonized World Soil Database v1.2 (2014).

39. Olson, D. M. & Dinerstein, E. The global 200: a representation approach to conserving the earth's most biologically valuable ecoregions. *Conserv. Biol.* **12**, 502–515 (1998).

40. Jenkinson, D. S., Poulton, P. R. & Bryant, C. The turnover of organic carbon in subsoils. Part 1. Natural and bomb radiocarbon in soil profiles from the Rothamsted long-term field experiments. *Eur. J. Soil Sci.* **59**, 391–399, DOI: 10.1111/j.1365-2389.2008.01025.x (2008).
